# Lack of Dose- and Time-Dependent Effects of Aflatoxin B1 on Gene Expression and Enzymes Associated with Lipid Peroxidation and the Glutathione Redox System in Chicken

**DOI:** 10.3390/toxins12020084

**Published:** 2020-01-26

**Authors:** Benjámin Kövesi, Mátyás Cserháti, Márta Erdélyi, Erika Zándoki, Miklós Mézes, Krisztián Balogh

**Affiliations:** 1 Department of Nutrition, Szent István University, H-2103 Gödöllő, Hungary; benjamin.kovesi@gmail.com (B.K.); Erdelyi.Marta@mkk.szie.hu (M.E.); Balogh.Krisztian@mkk.szie.hu (K.B.); 2 Department of Environmental Safety and Ecotoxicology, Szent István University, H-2103 Gödöllő, Hungary; Cserhati.Matyas@mkk.szie.hu; 3 MTA-KE-SZIE Mycotoxins in the Food Chain Research Group, Kaposvár University, H-7400 Kaposvár, Hungary; Zandoki.Erika@mkk.szie.hu

**Keywords:** aflatoxin B1, glutathione redox system, broiler chicken, lipid peroxidation, gene expression

## Abstract

Effects of aflatoxin B1 (AFB1) on lipid peroxidation and glutathione system were investigated in chicken liver. In a three-week feeding trial, different doses (<1.0 μg/kg (control diet), 17.0 µg (diet A1), 92.0 µg (diet A2), and 182.0 µg (diet A3) AFB1 kg/feed) were used. Markers of lipid peroxidation, conjugated dienes and trienes showed higher values in A3, while amounts of thiobarbituric acid reactive substances were increased in the A1 group at day 21. Glutathione content was lower at day 14 in Group A2. Glutathione peroxidase 4 activity was increased at days 7 and 21 in the A3 group but reduced in the A2 and A3 groups at day 14. The *GPX4* gene was downregulated at day 7 in the A2 group, but overregulated at days 14 and 21, and at day 14 in the A3 group. *GSS* was downregulated at day 14 in the A1 group but overregulated at day 21 in A1 and A2 groups. *GSR* was downregulated at days 7 and 21 in all treatment groups, but on day 14, induction was observed in the A3 group. The results indicated that AFB1 did not induce dose- or time-dependent effects on the glutathione redox system and its encoding genes at the dose range used, which means that oxidative stress is not the primary effect of AFB1 toxicity.

## 1. Introduction

Aflatoxins (AFs) are secondary metabolites of the *Aspergillus* species of fungi, which are generally contaminate tropical and subtropical food and feedstuffs [1]. However, due to climate change, their occurrence in temperate climates should be taken into account not only during storage but also on the field [2,3]. Climate change usually causes drought stress in fungi, and stress-responding pathways can stimulate the AF production of *Aspergillus flavus* [4].

AF contamination of poultry feed causes poor growth rate, liver and kidney damage, immunosuppression or even mortality [5,6]. AFs cause changes in the expression of genes encoding enzymes required for energy production, fatty acid metabolism and antioxidant defense [7,8,9,10]. Moreover, AFs, mainly aflatoxin B1 (AFB1) and their metabolites, may accumulate in edible products such as meat and eggs, which suggests public health concerns [11].

Chickens are found to be comparatively resistant to AFs [12] due to lower AFB1-8,9-exo-epoxide formation in microsomes, and lower formation of aflatoxicol in the cytosol [13]. Arafa et al. [12] reported that AFB1 at 0.7 mg kg/feed had negative effects on the growth rate of turkey poults but did not affect chickens. In addition, it was shown that a diet contaminated with 400 µg/kg AFB1 had marked effects on the relative weight of liver and bodyweight of turkey, but no alterations were found in chicken at this concentration [14].

Among the well-known AFs, AFB1 is the most biologically active form and is regarded as the most toxic one [15]. In fact, the native form of AFB1 is not really toxic, but after absorption, it is metabolized in the hepatocytes by phase I xenobiotic transforming enzymes into a highly reactive exo-AFB1-8,9 epoxide, which interacts with nucleotides and proteins [16] and is considered to have a primary role in the carcinogenic and mutagenic effects of AFB1 [17,18]. The International Agency for Research on Cancer classified AFB1 as a Group 1 human carcinogen [19].

It was described by *in vitro* [6,20,21] and *in vivo* [22,23] studies that AFB1 provokes reactive oxygen substances (ROS) formation and causes oxidative stress as one of the main causes of its toxic effects. It has also been reported that AFB1 alters intracellular antioxidant mechanisms, namely gene expression and protein synthesis of Nrf2, a redox sensitive regulator of the antioxidant response element (ARE) gene cluster, and consequently, it controls the synthesis of superoxide dismutase (SOD), glutathione peroxidase (GPx), glutathione synthetase (GS), glutathione reductase (GR), and catalase (CAT) [6,9,21]. Low activity of antioxidant enzymes and the decreasing level of glutathione (GSH) [24,25] consequently provoke oxidative stress due to the imbalance between oxidants and antioxidants [26,27,28].

Increasing formation of ROS causes activation of the antioxidant defense, as it is defined in the hierarchical model of oxidative stress [29]. The Keap1-Nrf2-ARE redox-sensitive pathway regulates the expression of antioxidant genes, and it is responsible for GSH biosynthesis. Under normal conditions, Keap1 negatively controls the Nrf2 transcription protein. As the effect of oxidation or alkylation, cysteine sites in Keap1 go under conformational changes, which lead to the accumulation of Nrf2 that is able to reach the nucleus, where it can bind to ARE and might induce the transcription of numerous of cytoprotective genes [30,31].

In this study, the changes in lipid peroxidation and glutathione redox system and expression of its regulatory genes were determined in liver. It is well known, that liver has high importance in xenobiotic transformation, and it is the primary site of GSH synthesis [32]. Within the biological antioxidant defense system, glutathione redox parameters were selected to be studied due to their central role in the antioxidant defense [33].

The purpose of the study is to investigate the effects of different doses of AFB1, based on the EU regulatory limit (20 µg/kg complete feed; Commission Regulation 574/2011) and on the results of the latest worldwide survey, which showed that the highest AFB1 contamination occurring in feed commodities in Europe was 176 µg/kg [34]. Thus, the dose-range was selected based on the contamination levels in poultry practice.

There are numerous studies about the effect of AFB1 on production traits and lipid peroxidation and antioxidant defense, but in those, mostly a single high dose of the mycotoxin was used, which has toxicological importance but did not show dose- or time-dependent effects. Our purpose is to investigate changes in the antioxidant response at gene expression and protein synthesis levels, which have not been published previously, in particular not in a dose- and time-dependent manner.

## 2. Results

The measured AFB1 content of the control diet was <1.0 μg/kg. Three experimental diets were artificially contaminated with AFB1 containing 17.0 μg/kg (Group A1); 92.0 μg/kg (Group A2), and 182.0 μg/kg (Group A3), respectively.

During the three-week-long experimental period, no mortality was observed in the experimental groups. Average calculated individual daily feed intake during the entire period did not differ among the experimental groups (C: 108.9 g/day; A1: 118.5/g day; A2: 114.7 g/day, and A3: 109.9 g/day, respectively), which means that no feed refusal was observed. The difference of the bodyweight of the birds was statistically significant only in Group A2 on days 7 and 14 as compared to the control (Table 1), but the differences were moderate (4.10% and 6.27%), and there was an initial 2.32% difference on day 0 between the two groups. No statistically significant difference was found in relative liver weight among the experimental groups during the trial (data not shown).

The conjugated diene level, as initiation phase marker of lipid peroxidation, showed significant difference as compared to control only in Group A2 after 14 days of AFB1 exposure (Table 2). The level of conjugated trienes also showed a significant difference on day 14 of exposure in Groups A2 and A3 as compared to the control (Table 2).

Termination marker of lipid peroxidation, the concentration of thiobarbituric acid reactive substances (TBARS), expressed as malondialdehyde (MDA), was significantly lower in Groups A1 and A3 after 7 days of AFB1 exposure as compared to the control. However, after 14 days of exposure, the MDA level was significantly lower in Groups A2 and A3 as compared to the control, while after 21 days of AFB1 exposure, higher MDA value was found in Group A1 as compared to the control (Table 2).

Reduced glutathione content of the liver homogenate was significantly lower only in Group A2, as compared to the control at day 14 of exposure (Table 3). 

GPx activity was significantly higher on day 7 of AFB1 exposure in Group A3 as compared to the control. However, significantly lower values were measured in Group A2 and A3 on day 14 as compared to the control. One week later, on day 21, significantly higher values were found only in Group A3 as compared to the control (Table 3). 

Relative expression of the *GPx4* gene was significantly lower in Group A2 than in the control on day 7 of AFB1 exposure. Later, on day 14, significantly higher values were observed in Groups A2 and A3, while in Group A2, significantly higher values were observed even on day 21 as compared to the control (Table 4).

*GSS* gene expression was significantly lower in Group A1 on day 14 and on day 21, while significantly higher values were measured in Groups A1 and A2 than in the control (Table 4). 

Relative gene expression of *GSR* was significantly lower in all treatment groups than in the control on day 7 of exposure, while on day 14, significantly higher values were measured only in Group A3. On day 21, significantly lower values were observed in *GSR* gene expression as the effect of AFB1 exposure in all treatment groups as compared to the control (Table 4). 

## 3. Discussion

The purpose of this study was to investigate the dose- and time-dependent effects of AFB1 on lipid peroxidation and antioxidant defense, at both gene expression and protein synthesis levels. The selected dose range was based on the regulatory limit in the EU (20 µg/kg complete feed; Commission Regulation 574/2011), and on the highest AFB1 contamination level of feed commodities recently found in Europe, which was 176 µg/kg [34].

The results revealed that AFB1 contaminated diets did not cause feed refusal, which was controversial with the results of a previous experiment [10], where marked feed refusal was found using 250 μg/kg feed. The reason might be the lower dose used in the present trial. 

Body weight was lower in the group exposed to the medium dose (five-times higher than the EU limit value), but at the highest (10x) dose, no significant difference was found. The same phenomenon, namely, a more pronounced effect of a low dose than a higher one, is known in poultry in case of aflatoxicosis [35]. There was no significant difference in relative liver weight, even at the highest dose (182 µg AFB1/kg feed) applied, which can be supported by other studies where significant differences in relative liver weight were found only at dose of AFB1 higher than 500 µg/kg feed [36,37].

Initiation phase markers of lipid peroxidation, conjugated dienes and trienes, showed minor differences among the groups during the trial. Statistically significant differences were observed only on day 14 of exposure in Group A2 for conjugated dienes, and in Groups A2 and A3, in which the levels of conjugated trienes were significantly higher than in the control. These results mean that AFB1 in the dose range applied caused measurable oxygen free radical formation only after a longer period (two weeks) of exposure, but even then, the initiation phase of lipid peroxidation was moderate and without biological relevance.

The termination marker of lipid peroxidation, TBARS, showed dual responses—lower values were found up to 14^th^ days of exposure and a slight increase thereafter, but only in the highest dose group. This result supports our previous explanation that AFB1 in the dose range applied caused only a moderate level of oxygen free radical formation in the liver of chicken. TBARS is widely used as a marker of lipid peroxidation and its high level reflects oxidative stress [38]. However, in the present study, only minor changes were observed, which suggests that the applied doses did not cause oxidative stress. This finding is contradictory with some previous studies with broiler chickens exposed to AFB1 where marked induction of lipid peroxidation was found, but in those studies, higher doses and longer periods of exposure, such as 1000 μg/kg, 4 weeks [39] or 2000 μg/kg, 6 weeks [40], were applied.

It is well known that the glutathione redox system has an important role in antioxidant defense against the effects of oxygen-free radicals as they neutralize hydrogen peroxide and lipid peroxides [41]. GSH content has been decreased significantly in the liver, which is the main site of its biosynthesis [32], as was found in Group A2 after 14 days of AFB1 exposure. However, the same effect was found in a previous study at a higher dose (3000 μg/kg feed for 21 days) of exposure [42]. GSH depletion was found after 14 days of AFB1 exposure, which suggests a lower rate of reduction of glutathione disulphide to GSH or de novo GSH synthesis. GPx4 activity showed similar changes as GSH. The enzyme activity in the liver homogenates was higher after 7 days of exposure as the effect of the highest dose (Group A3), which suggests an early antioxidant response to AFB1-induced oxidative stress, possibly by post-translational modification of GPx protein. Later, on day 14, lower values were measured in Groups A2 and A3, and on day 21 in Group A3. These results suggested that the lower the AFB1 dose applied, the later the response, but the response to the higher dose was not adequate for a long period. In a previous study, marked reduction of GPx activity was reported as an early response, however, at a different dose (2000 μg/kg) of exposure [40], which suggested that the dose applied in present study did not cause the level of oxygen free radical formation that is required for the induction of gene expression and/or post-translational modification of the enzyme protein.

Changes in the expression of the investigated genes suggest that AFB1 exposure activates the synthesis and recycling of the components of the glutathione redox system [43]. Muhammad et al. [44] reported that AFB1 exposure caused downregulation in Nrf2 at mRNA and protein levels and in expression of xenobiotic transformation phase II genes, such as GST, in broiler chicken. Low Nrf2 expression, as the effect of AFB1 exposure, may cause downregulation of the expression of antioxidant gene clusters such as *SOD*, *GPX* or *GST*, as it was found in chicken liver [7,8,23,45]. Among the GPx isoenzymes, glutathione peroxidase 4 (*GPX4*) gene expression has primary importance in the antioxidant defense of the avian species [46], while in mammals, *GPX1* plays the major role [47].

In the present study, gene expression of *GPX4* showed dual responses. After 7 days of exposure, significantly lower values were observed in Group A2, while as the effect of the lowest and highest doses (Groups A1 and A3), nearly control expression levels were measured. Later, on day 14 of AFB1 exposure, an induction was observed in Groups A2 and A3. Furthermore, in Group A2, this induction was also observed after 3 weeks of AFB1 exposure. However, the changes in gene expression were not followed by the same tendencies in GPx activity, which means that changes in gene expression were not reflected in protein synthesis and enzyme activity. In the case of the *GR* gene expression, a dual response was also revealed. After 7 days of AFB1 exposure, downregulation was observed in all treatment groups, which then turned to upregulation after 14 days in case of the medium (A2) and high (A3) dose groups, and on day 21 of exposure, downregulation was observed in all treatment groups again. Expression of the *GS* gene showed only minor changes. Downregulation was observed after 14 days of AFB1 exposure in Groups A1 and A2, which turned into induction after 21 days of exposure. The alterations in the expression of *GR* and *GS* genes may explain the changes in GSH level, but the dose-dependent differences at different samplings suggest that the antioxidant gene cluster is controlled by transcription factors such as Nrf2.

According to the results during the three-week-long experiment, none or only mild oxidative stress occurred, which can be explained by the hierarchical model of oxidative stress [28]. The possible cause of lack or mild oxidative stress would be that chickens are considered relatively resistant to AFs [12,13]. This is probably due to the lower level of reactive AFB1-8,9-exo-epoxide formation, which is required for further steps to oxidative stress. According to the above-mentioned model, the mild oxidative stress is associated with induced expression of antioxidant enzymes via the regulation by transcription factors, Keap1 and Nrf2. As the AFB1 dose range applied did not cause real activation of the antioxidant system and induction of gene expression, longer exposure and/or higher ROS levels are thought to be required for such changes. Therefore, we hypothesize that a critical level of ROS is required for the activation of Nrf2, after redox-sensitive conformational changes of its inhibitory protein, Keap1. Consequently, the newly synthesized Nrf2 can accumulate and translocate to the nucleus only after those changes [29]. The results of the present study has revealed that AFB1 at the dose range used and during the period of the trial did not cause such dose- or time-dependent ROS formation, which is required to reach the critical level for induction of mild oxidative stress. This hypothesis is supported by the results of our study, in which lipid peroxidation and activation of the Nrf2-ARE pathway showed only tendencies of slight changes, but the differences were statistically significant only in certain cases, and at different doses of exposure.

In conclusion, the results suggest that AFB1 induces mild oxidative stress, but this response is not dose- or time-dependent and the antioxidant system is not activated. It means that oxidative stress is not the main cause, but possibly only a consequence of its toxicity in the dose-range applied. Antioxidant response at either gene expression or protein synthesis levels to AFB1-induced oxidative stress suggests that a low level of oxidative stress was induced, but high doses might impaired the antioxidant response, which explains the dose- and time-independent changes.

## 4. Materials and Methods

### 4.1. Animals and Experimental Design

A total of 78 Cobb 540 broiler chickens obtained from a commercial hatchery (Babádi Hatchery Ltd., Felsőbabád, Hungary) were reared according to the standard Cobb technology up to 21 days of age. At 21 days of age, the animals in the same room were divided into four groups (*n* = 18, two replicates (*n* = 9) each) and additional 6 birds served as absolute control. Housing conditions were deep litter and natural light regimen (12 L/12 D). Before starting the feeding of AFB1-contaminated diets, 12 h of feed deprivation was applied. Nutrient content of the basal diet (chicken grower complete feed; Vitafort Ltd., Dabas, Hungary) met the requirements for broiler chickens [48] without containing mycotoxin binder and coccidiostat.

Before allocating the animals to the treatment groups, six randomly selected animals were euthanized at day 0 as absolute control. Six birds from each group (3 per replicate: control and Groups A1, A2 and A3) were sampled on days 7, 14 and 21 of the experiment. Post mortem, liver samples were taken and stored at −70 °C until biochemical analysis, while for gene expression analyses, small portions were taken into liquid nitrogen and stored at −70 °C until analyses.

AF was produced in corn artificially infected with an *Aspergillus flavus* strain isolated by Dobolyi et al. [2]. The strain was identified and deposited in the Microbiological Collection of the University of Szeged (SZMC) with the accession number SZMC 20750. The measured AFB1 concentration of the infected corn substrate was 4.694 mg/kg dry matter. 

Basal diet was contaminated with mixing an appropriate amount of AFs containing corn substrate. The measured AF content of the diets is given in Table 5.

AF content of the experimentally contaminated feeds was analyzed from three replicate samples (20 g each), which were taken from five different points of batch (10 g each) and thoroughly homogenized before preparing the analytical samples. Feed samples were analyzed after extraction with acetonitrile: water (9:1, *v/v*) immune-affinity clean-up was made with Aflaprep^®^ column (R-Biopharm, Darmstadt) and after reversed phase isocratic (acetonitrile:methanol:water; 8:27:65, *v/v/v*) HPLC method with fluorescence detection [49]. LOQ of the determination was 0.1 μg/kg for AFB1, 0.2 μg/kg for AFB2, 0.5 μg/kg for AFG1, and 0.5 μg/kg for AFG2, respectively.

### 4.2. Ethical Issues

The experiment was carried out according to the Hungarian Animal Protection Act, in compliance with the relevant EU rules. The experimental protocol was authorized by the Department of Food Chain Safety, Land Register, Plant and Soil Protection and Forestry of the Pest County Government Office (Hungary) with a permission number PE/EA/1964-7/2017.

### 4.3. Measurement of Feed Intake, Mortality, Body Weight, Liver Weight, and Relative Liver Weight

Feed intake was measured in each group daily. Mortality was checked daily in each experimental group. Bodyweight (grams) was measured at each sampling. After cervical dislocation and bleeding, liver weight (grams) was measured at necropsy after removal of the gall bladder, and relative liver weight was calculated as liver weight/100 g body weight.

### 4.4. Biochemical Analyses

Conjugated dienes (CD) and conjugated trienes (CT), biomarkers of the initial phase of lipid peroxidation, were determined with the absorbance of samples at 232 and 268 nm after extraction in 2,2,4-trimethylpentane [50]. TBARS, as a marker of the termination phase of lipid peroxidation, was determined based on complex formation with 2-thiobarbituric acid at high temperature (100 °C) and acidic pH [51] and expressed as malondialdehyde (MDA), using 1,1,3,3-tetrathoxypropane as standard.

Determination of reduced glutathione (GSH) concentration was performed with the method of Rahman et al. [52] based on the color complex formation of non-protein sulfhydryl groups with 5,5′-dithiobis (2-nitrobensoic acid). The activity of glutathione peroxidase (GPx) was analyzed as described by Lawrence and Burk [53] using cumene hydroperoxide as substrate for GPx4, and GSH as co-substrate, and expressed as units, which means 1 nmol GSH oxidation per minute at 25 °C. TBARS were determined in native 1:9 liver homogenate in isotonic saline (0.65% *w/v* NaCl), while GSH content and GPx activity were measured in its 10,000 g supernatant fraction. GSH content and GPx activity were calculated to protein content of the supernatant fraction of homogenate, which was determined according to Lowry et al. [54].

### 4.5. RNA Isolation, Reverse Transcription and qPCR

Total RNA was extracted by Trizol reagent (Molecular Research Centre, Cincinnati, OH, USA) from 10 mg liver tissue homogenate based on the instructions of the manufacturer. RNA samples were treated with DNase I (Thermo Fisher Scientific, San Jose, CA, USA) to avoid genomic DNA contamination. Agarose gel electrophoresis was performed to check the quality and integrity of RNA, and an absorption ratio 260:280 nm higher than 2.0 was accepted. cDNA production was implemented with RevertAID Reverse transcriptase (Thermo Fisher Scientific, San Jose, CA, USA) based on a standard protocol. The primers used for the quantification of relative mRNA transcription of *GPX4*, *GSS,* and *GSR* and the control gene glyceraldehyde 3-phosphate dehydrogenase (*GAPDH*) were as described previously [5]. *GAPDH* can be used as a control because some previous studies with mycotoxins in broiler chickens [7,10] did not found interaction with its relative expression in oxidative stress.

### 4.6. Statistical Analyses

Normality of distribution and homogeneity of variance were tested with the Shapiro–Wilk test and the Bartlett and Browne–Forsythe test, respectively. Data with these conditions were analyzed by one-way ANOVA. Significance of differences between groups was evaluated using post-hoc Tukey test (*p* < 0.05). Analyses were performed with GraphPad Prism 7.0 (GraphPad Software, San Diego, CA, USA). All data are presented as mean ± standard deviation (SD).

## Figures and Tables

**Table 1:** Effect of aflatoxin B1 (AFB1) treatment on body weight (g) of chickens (mean ± SD; n = 6).

Group	Day 0	Day 7	Day 14	Day 21
Control	614.6 ± 84.0	995.3 ± 143.7^a^	1462.7 ± 232.5^a^	1988.9 ± 329.0
A1	610.8 ± 84.5	961.0 ± 134.0^ab^	1435.8 ± 182.9^ab^	1938.8 ± 233.0
A2	600.3 ± 67.2	954.4 ± 120.8^b^	1371.0 ± 185.6^b^	1851.3 ± 204.7
A3	606.5 ± 70.8	959.6 ± 116.1^ab^	1396.2 ± 170.8^ab^	1874.2 ± 226.1

^a,b^ Different superscripts in the same column mean significant difference as compared to the control at *p* < 0.05 level, whrere ^a^ was the control.

**Table 2:** Effect of AFB1 treatment on parameters of lipid peroxidation in crude liver homogenates (mean ± SD; *n* = 6).

**Conjugated dienes (OD 232 nm)**				
	Day 0	Day 7	Day 14	Day 21
Control	0.312 ± 0.012	0.289 ± 0.011	0.269 ± 0.013^a^	0.289 ± 0.018
A1		0.300 ± 0.028	0.280 ± 0.021^ab^	0.311 ± 0.039
A2		0.284 ± 0.014	0.313 ± 0.027^b^	0.307 ± 0.050
A3		0.275 ± 0.012	0.304 ± 0.024^ab^	0.282 ± 0.028
**Conjugated trienes (OD 268 nm)**				
	Day 0	Day 7	Day 14	Day 21
Control	0.175 ± 0.006	0.162 ± 0.009	0.145 ± 0.008^a^	0.157 ± 0.009
A1		0.165 ± 0.017	0.151 ± 0.011^ab^	0.163 ± 0.011
A2		0.153 ± 0.007	0.175 ± 0.014^c^	0.157 ± 0.010
A3		0.151 ± 0.006	0.163 ± 0.009^bc^	0.147 ± 0.011
**TBARS (malondialdehyde μmol/g wet weight tissue)**				
	Day 0	Day 7	Day 14	Day 21
Control	58.19 ± 11.10	71.66 ± 4.34^b^	63.46 ± 12.21^b^	30.60 ± 4.48^a^
A1		46.39 ± 6.38^a^	50.24 ± 13.45^ab^	51.79 ± 7.42^b^
A2		61.27 ± 8.33^ab^	34.28 ± 10.89^a^	25.07 ± 10.76^a^
A3		47.71 ± 17.50^a^	42.40 ± 7.49^a^	36.88 ± 9.80^a^

^a,b,c^ Different superscripts in the same column mean significant difference as compared to the control at *p* < 0.05 level, where ^a^ was the control, and ^c^ means significantl difference to all other groups.

**Table 3:** Effect AFB1 treatment on the amount/activity of glutathione redox system of 10,000 g supernatant fraction of liver homogenates (mean ± SD; *n* = 6).

**Reduced glutathione** (μmol/g protein content)				
	Day 0	Day 7	Day 14	Day 21
Control	4.68 ± 0.60	4.68 ± 0.91	5.02 ± 0.64^b^	3.92 ± 0.64
A1		4.66 ± 0.46	5.12 ± 0.93^b^	4.19 ± 0.56
A2		5.45 ± 0.64	3.76 ± 0.80^a^	4.32 ± 0.61
A3		5.27 ± 1.13	3.98 ± 0.28^ab^	4.94 ± 0.85
**Glutathione peroxidase** (U/g protein content)				
	Day 0	Day 7	Day 14	Day 21
Control	4.18 ± 2.13	3.96 ± 0.74^a^	4.99 ± 0.54^b^	3.31± 0.68^a^
A1		4.00 ± 0.53^a^	5.41 ± 0.74^b^	3.60 ± 0.67^ab^
A2		5.10 ± 0.51^ab^	3.24 ± 0.63^a^	4.39 ± 0.63^ab^
A3		5.19 ± 1.04^b^	3.13 ± 0.44^a^	4.67 ± 0.71^b^

^a, b^ Different superscripts in the same column mean significant difference as compared to the control at *p* < 0.05 level, where ^a^ was the control

**Table 4:** Effect of AFB1 treatment on the relative expression of *GPX4*, *GSR* and *GSS* genes in liver of broiler chicken (mean ± SD; *n* = 6 in a pool, equal amounts of cDNA per individual).

**Glutathione peroxidase 4 (*GPX4*)**				
	Day 0	Day 7	Day 14	Day 21
Control	1.00 ± 0.03	0.93 ± 0.05^bc^	0.78 ± 0.05^a^	1.80 ± 0.09^a^
A1		0.85 ± 0.03^ab^	0.79 ± 0.08^a^	1.82 ± 0.06^a^
A2		0.79 ± 0.04^a^	1.10 ± 0.02^b^	2.26 ± 0.08^b^
A3		0.99 ± 0.06^c^	1.06 ± 0.07^b^	1.89 ± 0.19^a^
**Glutathione synthetase (*GSS*)**				
	Day 0	Day 7	Day 14	Day 21
Control	1.04 ± 0.07	1.22 ± 0.06	1.42 ± 0.08^b^	1.35 ± 0.12^a^
A1		1.10 ± 0.09	1.13 ± 0.15^a^	1.54 ± 0.19^b^
A2		1.20 ± 0.14	1.28 ± 0.11^ab^	1.57 ± 0.11^b^
A3		1.07 ± 0.12	1.41 ± 0.10^b^	1.39 ± 0.21^ab^
**Glutathione reductase (*GSR*)**				
	Day 0	Day 7	Day 14	Day 21
Control	1.00 ± 0.04	1.19 ± 0.14^b^	1.05 ± 0.08^ab^	2.23 ± 0.09^b^
A1		0.96 ± 0.10^a^	0.91 ± 0.07^a^	1.87 ± 0.13^a^
A2		0.92 ± 0.07^a^	1.16 ± 0.14^bc^	1.88 ± 0.10^a^
A3		1.00 ± 0.07^a^	1.27 ± 0.06^c^	1.76 ± 0.12^a^

^a,b,c^ Different superscripts in the same column mean significant difference as compared to control at *p* < 0.05 level, where ^a^ was the control, ^ab^ means not significant difference to control, ^c^ means significantl difference to other groups, and ^bc^ means not signficiant difference to groups marked with ^c^, only to control

**Table 5:** Aflatoxin content of experimental diets (μg/kg).

Diet	AFB1	AFB2	AFG1	AFG2
Control	<1.0	<1.0	<0.5	<0.5
A1	17.0	<1.0	<0.5	<0.5
A2	92.0	6.0	<0.5	<0.5
A3	182.0	12.0	<0.5	<0.5
